# The association between the recurrence of solitary non-muscle invasive bladder cancer and tumor infiltrating lymphocytes

**DOI:** 10.3325/cmj.2012.53.598

**Published:** 2012-12

**Authors:** Kristian Krpina, Emina Babarović, Gordana Đorđević, Željko Fučkar, Nives Jonjić

**Affiliations:** 1 Department of Urology, Clinical Hospital Center Rijeka, Rijeka, Croatia; 2 Department of Pathology, School of Medicine, University of Rijeka, Rijeka, Croatia

## Abstract

**Aim:**

To evaluate whether tumor infiltrating lymphocytes (TIL) in biopsy specimens are associated with the clinical outcome of non-muscle invasive bladder cancer.

**Methods:**

We retrieved tumor specimens from 115 patients with solitary papillary non-muscle invasive bladder cancer treated between 1996 and 2006 and constructed tissue microarrays. Patients were divided in two groups: those with recurrent disease (N = 69) and those without recurrent disease (N = 46) during the follow up of minimum 5 years. All patients were treated with initial transurethral resection and none received adjuvant therapy. Immunhistochemical staining was performed with anti-CD3, CD4, CD8, and Granzyme B (GrB). The CD4+:CD8+ and GrB+:CD8 ratios were determined.

**Results:**

Tumor infiltrating lymphocytes were predominantly observed within cancer stroma, and only rare individual cells were observed intraepithelially. The group without recurrent disease had lower levels of CD3+ and CD8+ lymphocytes than the group with recurrent disease (*P* = 0.0001, *P* = 0.0002, respectively). The CD4+:GrB+ and GrB+:CD8+ ratios were significantly higher in patients without recurrent disease (*P* = 0.0002, *P* = 0.039, respectively).

**Conclusion:**

This study revealed a possible connection between TIL number and bladder cancer recurrence. TIL subset ratio showed different patterns in recurrent and non-recurrent tumors, which is why it could become a useful a prognostic clinical index if our findings are confirmed in randomized trials.

Bladder cancer is the most common malignancy of the urinary tract (1), with non-muscle invasive bladder carcinoma being its most frequent type (75%-85% of patients) (NMIBC, stages pTa, pT1, pTis). These tumors form a very heterogeneous group, with various clinical outcomes. The main clinical feature of bladder cancer is the high recurrence rate. In fact, 70% of the patients treated by transurethral resection (TUR) experience a relapse of the underlying disease, while in 15%-25% the disease over time turns into muscle-invasive cancer (2). Given that multiple different therapeutic approaches are available, assessing the risk of tumor recurrence and progression is the key task in developing individualized and accurate adjuvant treatment (3,4).

Urinary bladder carcinoma is the only malignant neoplasm whose treatment often includes immunotherapy. The recurrence rate and risk of progression to muscle-invasive disease in patients with non-muscle-invasive urothelial carcinomas has been reduced by intravesical instillation of Bacille Calmette-Guerin (BCG) (5,6). Although some variables that predict the recurrence have been used to identify patients who require adjuvant therapy after TUR, additional reliable markers for disease progression and recurrence are needed (7,8). BCG induces a massive influx of cytokines and inflammatory cells into the bladder wall and lumen, and its antitumor activity may be generally attributed to potent activation of cellular immunity (9,10). Tumor-infiltrating immune cells are thought to play an important role in preventing disease progression and therapeutic efficacy in other cancers (11-15). Thus, the aim of the present study was to determine the association of tumor infiltrating lymphocytes and solitary papillary NMIBC in biopsy specimens obtained from patients grouped according to development of the recurrent disease. We hypothesize that such investigation could be helpful in identifying patients with recurrent tumors who require adjuvant treatment.

## Patients and methods

### Clinicopathological data

During 2010 and 2011, we collected data on all 115 patients with solitary papillary NMIBC who underwent initial transurethral resection, which is a standard treatment regimen at the Department of Urology, Rijeka University Hospital Center in Rijeka, between 1996 and 2006. The data were gathered from hospital charts and medical histories. We included only the patients operated on till 2006 to ensure the follow-up of a minimum 5 years.

None of the patients received adjuvant chemotherapy, immunotherapy, or any other medical intervention after the initial TUR. All patients with multiple tumors and patients with a solid or flat aspect were excluded from this study. The study was approved by the University of Rijeka Ethics Committee and all patients signed informed consent.

All sections were reviewed to confirm the original diagnosis and were then staged according to the 2002 American Joint Committee on Cancer (16) and graded according to the 2004 World Health Organization classification system (17) by an expert urologic pathologist (GĐ). All tumors were classified as papillary urothelial neoplasm of low malignant potential or low-grade papillary urothelial carcinoma. Patients were divided in two groups: patients who developed recurrent disease (study group, N = 69) and patients who did not develop recurrent disease during the follow-up of a minimum 5 years (control group, N = 46). The follow-up of patients was scheduled at control cystoscopy for every 3 months during the first two years after TUR, and after that biannually. Recurrence was defined as a written description of the recurrent tumor at any control cystoscopy 6 months after the operation, localized away from the primary tumor bed and the area of the initial resection to exclude all possible residual tumor masses.

### Tissue microarray construction (TMA)

On hematoxylin and eosin stained tumor sections, we marked the areas with the most pronounced inflammatory infiltration. On each slide, an expert uropathologist chose three “hot-spots,” which were then analyzed for TIL count. These “hot-spots” were defined as the areas of the most prominent peritumoral mononuclear infiltration (18). Paraffin blocks were available for all 115 cases, and tissue microarrays (TMA) were constructed by using a manual tissue arrayer (Alphelys, Plaisir, France). Three tissue cores, each 1 mm in diameter, were placed into a recipient paraffin block. Normal liver tissue was used for orientation. The cores were spaced at intervals of 0.5 mm along the x- and y-axis. One section from each TMA block was stained with hematoxylin and eosin to confirm the presence of tumor tissue. Serial sections were cut from TMA blocks for immunohistochemical staining. Three to four micrometers-thick sections were placed on adhesive glass slides (Capillary Gap Microscope Slides, 75 μm, Code S2024, DakoCytomation, Glostrup, Denmark), left to dry in the oven at 55°C overnight, and were deparaffinized and rehydrated. TMAs were created and interpreted by two expert uropathologists and one pathologist. The same three pathologists were involved in reading of the results and reached a consensus on all the results.

### Immunohistochemistry and evaluation of staining by computerized image analysis

Tumor samples were subjected to immunohistological analysis in a Dako Autostainer Plus (DakoCytomation Colorado Inc, Fort Collins, CO, USA) according to the manufacturer's protocol using Envision peroxidase procedure (ChemMate TM Envision HRP detection kit K5007, DakoCytomation) and different antibodies for immunohistochemical staining, including epitope retrieval, dilution, and incubation (Table 1).

**Table 1:** Antibodies used for immunohistochemical staining

Antigen	Catalog number	Species	Clone	Source	Epitope retrieval	Dilution	Incubation	Positive control
**CD 3**	M 7254	Mouse mAb	F7.2.38	DakoCytomation	Citrate buffer pH 6.0, microwave oven 25 min	1:100	30 min/RT*	appendix
**CD 4**	NCL-L-CD4-368	Mouse mAb	4B12	Novocastra	EDTA buffer pH 8, microwave oven 25 min	1:75	60 min/RT	tonsil
**CD 8**	M 7103	Mouse mAb	C8/144B	DakoCytomation	Tris/EDTA buffer pH 9.0 and water bath at 98°C for 15 min	1:100	30 min/RT	tonsil
**GrB**	M 7235	Mouse mAb	GrB-7	DakoCytomation	Tris/EDTA buffer pH 9.0, microwave oven 25 min	1:35	60 min/RT	tonsil

*RT – room temperature; GrB – Granzyme B.

All antibody-stained slides were scanned and analyzed with Alphelys Spot Browser 2 integrated system, using software-controlled (Alphelys Spot Browser 2.4.4.) stage positioning Nikon Eclipse 50i microscope-mounted 1360 × 1024 resolution Microvision CFW-1310C digital camera. The slides were scanned at 20 × magnification to identify the section area of the slide and then at 200 × magnification to create images for quantification and scoring positive cells. Positive cells were counted in the tumor stroma, lymphoid aggregates, and intraepithelially, and presented as absolute numbers and as a proportion of positive cells in the total inflammatory infiltrate. In statistical analysis, the mean value of immunohistochemical staining of all three tissue microarrays was used.

### T lymphocyte subpopulation analysis

In order to determine the exact level of immunobiological interactions in the bladder of these patients, we also used subset ratio analysis because it had been previously shown that this ratio more accurately represented the immunobiological status of the patient than the number of infiltrated cells (19-21).

### Statistical analysis

Statistical analysis was performed using SPSS 20 software (SPSS Inc., Chicago, IL, USA). The distribution of data was tested for normality using D’Agostino-Pearson test. Independent *t* test and Mann-Whitney U test were used to assess whether continuous variables differed significantly between categories, depending on data distribution.

## Results

The median patients’ age at diagnosis was 73 years (range 41-86), with a male-to-female ratio of 3.3:1, and a median follow-up period of 73.5 months (range 6-155). This range is so wide because all patients had a lifelong follow-up, but for the purposes of the study the follow-up duration in patients with recurrent disease was determined to be until the recurrence and in patients without recurrent disease a minimum of 5 years. The recurrence was observed in 69 patients (60%). Clinical and pathological characteristics were equally distributed, ie, 51% of patients had tumors larger than 3 cm and 53% of patients had pT1 stage (Table 2).

**Table 2:** Clinicopathologic characteristics of patients with non-muscle invasive bladder carcinoma*

	No. (%) of patients	
	with recurrent disease (n = 69)	without recurrent disease (n = 46)
**Sex:**		
male	52 (75)	36 (78)
female	17 (25)	10 (22)
**Age in years (median, range)**	74.4 (41-87)	71.8 (54-78)
**Tumor size:**		
>3 cm	40 (58)	19 (41)
≤3 cm	29 (42)	27 (59)
**Pathology:**		
PUNLMP	26 (38)	28 (61)
LGPUC	43 (62)	18 (39)
**Median follow-up (months)**	13	75

*Abbreviations: PUNLMP – papillary urothelial neoplasm of low malignant potential; LGPUC – low-grade papillary urothelial carcinoma.

**Figure 1:**
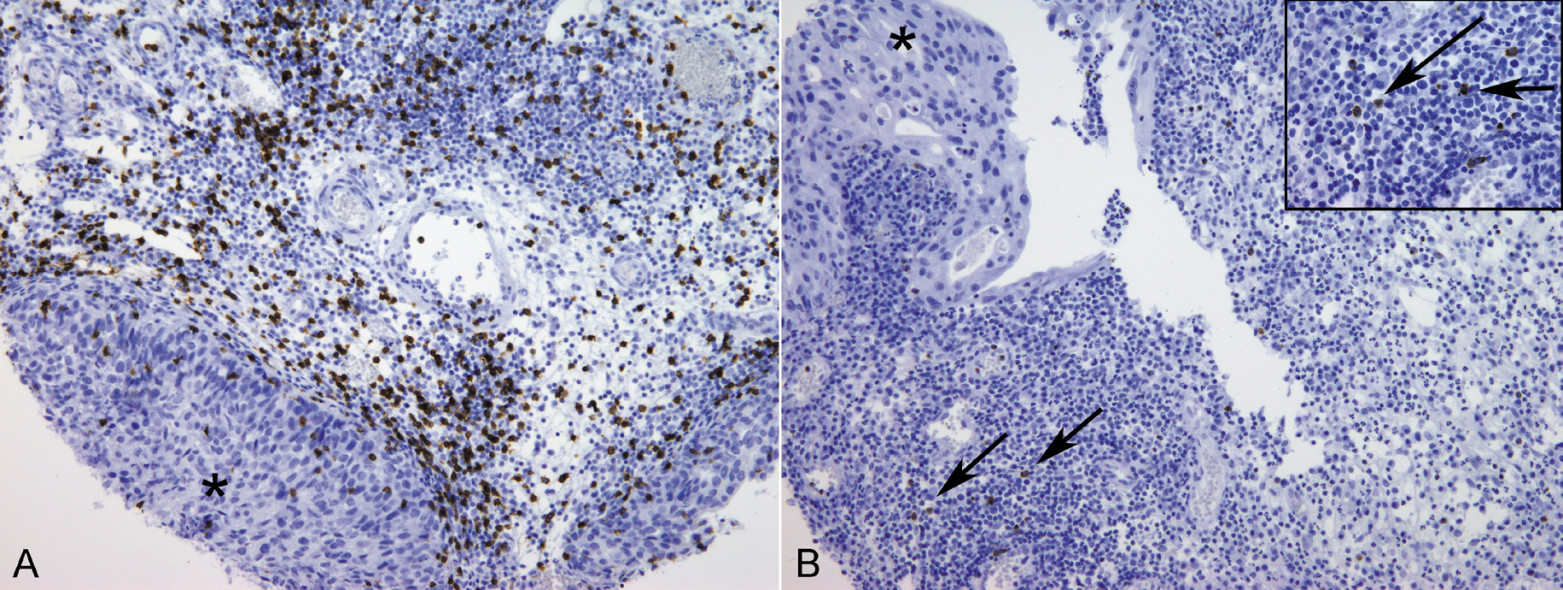
Immunohistochemical staining with anti-CD8 (**A**) and Granzyme B (**B**) in representative tumor samples of non-muscle invasive bladder cancer (original magnification 100 × ). (**A**) Abundant tumor infiltrating CD8 lymphocytes predominantly within the cancer stroma accompanied by rare individual cells within the epithelial part (indicated by asterisk) of the tumor. (**B**) Scatter single Granzyme B positive cells (arrows) in the tumor stroma and representative high-power (400 × magnification) image (upper panel).

Tumor infiltrating lymphocytes (TIL) CD3+, CD4+, CD8+, and GrB+ were predominantly observed within the cancer stroma, especially in the papillary-axis, underlying stroma, and lymphoid aggregates, and only rarely as individual cells within the epithelial part of the tumor (Figure 1).

We analyzed the number of CD3+, CD4+, CD8+, and GrB+ cells in each patient. The group without recurrence had significantly fewer infiltrating CD3+ and CD8+ T cells (*P* = 0.0001, *P* = 0.0002, respectively), but no significant differences in the number of CD4+ and GrB+ cells were observed between the groups (*P* = 0.561 [95% confidence interval -8-4], *P* = 0.654 [95% confidence interval -1-1], respectively) (Table 3).

**Table 3:** The number of tumor-infiltrating lymphocytes in the groups of patients with and without recurrent disease

Marker expression	Recurrent disease		*P*	95% confidence interval
	yes (n = 69)	no (n = 46)		
**CD 3 (**mean ± standard deviation, %)	46 ± 17	30 ± 14	0.0001*	10.13-22.21
**CD 4 (**median, range, %)	32 (2-67)	34.5 (8-61)	0.561^†^	-8-4
**CD 8** (median, range, %)	16 (1-32)	11.5 (1-32)	0.0002^†^	2-8
**GrB** (median, range, %)	3 (0-17)	3 (0-13)	0.654^†^	-1-1

*Independent *t* test.

†Mann- Whitney U test.

The ratio between different subpopulations of T lymphocytes was determined, eg, CD4+:CD8+ and GrB+:CD8+ cells. In the entire cohort of patients, the proportion of CD4+ cells was 2.69 times higher than that of CD8+ cells, and the proportion of CD8+ cells was 3.82 times higher than that of GrB+ cells. The proportion of CD4+:CD8 and GrB+:CD8 T cells was significantly higher in the group without recurrence than in the group with recurrence (*P* = 0.0002 [95% confidence interval -2.267 to -0.775], *P* = 0.039 [95% confidence interval -1.428 to -0.0037], respectively) (Table 4).

**Table 4:** The ratio between CD4+:CD8+ and Granzyme B+:CD8+ T lymphocytes in the groups of patients with and without recurrent disease (N = 115)

Ratio	Recurrent disease		P*	95% confidence interval
	yes (n = 69)	no (n = 46)		
**CD4:CD8**			**0.0002**	-2.267 to -0.775
median (range)	1.86 (0.06-39)	3.39 (0.45-14)		
mean ± standard deviation	2.69 ± 4.9	3.97 ± 2.87		
**GrB:CD8**			**0.039**	-0.1428 to -0.0037
median (range)	0.17 (0-1.17)	0.23 (0.04-3)		
mean ± standard deviation	0.26 ± 0.28	0.40 ± 0.5		

*Mann-Whitney U test; GrB – Granzyme B.

## Discussion

The present study found differences in the number of TILs between the group of patients with recurrence and the group of patients without recurrence at the time of initial TUR. Moreover, it found significantly higher levels of CD3+ and CD8+ TILs in the group of patients with recurrence, while this was not confirmed for CD4+ and GrB+ cells. Since no patient had received any other form of treatment, we assume that these results reflect the real immune status of patients at the time of the initial operation, which is directly dependent on the immunobiology and pathophysiology of the neoplastic disease.

Our findings on CD3+ TILs are similar to the results of Lipponen et al (22), who found that CD3+ T cells in NMIBC predicted the progression of tumors and the related short recurrence-free survival. They hypothesized that unfavorable prognosis related to the presence of TILs may be connected to the inhibitory mediators released by the tumor cells. These results are in contrast to the findings obtained by other authors, who reported good prognosis for the patients with higher number of TILs (23-25).

There are several possible explanations for the different literature findings regarding prognostic significance of TILs. One may be heterogeneity of the analyzed bladder cancers, since no distinction was made between the non-muscle and muscle invasive type. This is why the present study was conducted on a homogenous group of patients, ie, only those who exhibited a solitary NMIBC. Other explanations may be the use of different methodologies for TIL quantification and the lack of a different T cell subtypes analysis. According to our results, it was more appropriate to determine the percentage of particular lymphocyte subsets than their absolute numbers.

The percentage of CD4+ cells in our study was higher than that of CD8+, the CD4+:CD8+ ratio being 3:1, irrespective of the recurrence of the disease. The percentage of CD4+ TILs was practically the same for non-recurrent and recurrent disease. This finding suggests that the infiltration of CD4+ TIL cells to the tumor site occurs under similar conditions.

Tumor infiltrating CD4+ cells play a central role in immune protection through their production of cytokines and chemokines, thus orchestrating immune responses. However, the CD4+:CD8+ TIL ratio was significantly lower in the group with the recurrence, or more specifically, this group had a greater number of CD8+ TILs, thus indicating that CD8+ TILs could be associated with bladder cancer recurrence.

To the best of our knowledge, this report is the first to describe the CD4+:CD8+ ratio in bladder cancer. This ratio has already been confirmed as an immunobiological marker used for evaluation of the immune system status in patients with ovarian, colorectal, breast, and hepatocellular cancer (19,20,26,27).

In contrast to our study, Sharma et al (28) did not associate CD8+ TILs with disease-free survival and overall survival among patients with non/muscle invasive bladder cancer, although CD8+ TILs did predict the survival in muscle invasive urothelial carcinoma. On the other hand, Jacobs et al (29) found a significant increase in the number of peripheral blood CD8+ lymphocytes in patients with recurrent NMIBC. They hypothesized that these peripheral immune suppressive cells facilitated tumor recurrences or that tumor recurrences caused an increase in the peripheral immunosuppressive lymphocytes (29).

We also analyzed data on anti-tumor effector cells such as GRB+. As expected, the number of GrB+ cells was lower than that of CD8+ TILs in the entire patient group, although the GrB+:CD8+ ratio was significantly higher in the group with non-recurrence. This is consistent with the concept of apoptotic role of GrB cells. GrB is a serine proteinase contained in cytoplasmic granules of activated cytotoxic T lymphocytes and natural killer cells. In the effector-target cell interaction, it is exocytosed and delivered inside the cytosol of target cells, where it induces apoptosis via proteolysis of intracellular substrates (30). Since the levels of GrB+ TILs were similar in both patients with and without recurrence, the cytotoxic mechanism was probably not impaired. The cause of tumor recurrence may most likely be associated with immunosupression. It is well known that bladder cancer is an immunogenic malignancy, characterized by the presence of TILs, which are probably capable of detecting neoplastic transformation and eradicating tumors cells. On the other hand, tumor cells escape immune destruction, since immune system cells are known to play both protective and tumor-promoting roles during neoplastic transformation. Tumors undergo a process known as immunoediting, in which the tumor establishes a favorable microenviroment that enables it to avoid immune destruction (31). Patients with bladder cancer also exhibit a tumor-associated immunologic suppression, particularly evident as an impaired T-cell response. Indeed, bladder tumors have been shown to be infiltrated by T regulatory cells and to express high levels of inhibitory cytokines, which may in part explain the immune dysfunction (32,33). A mechanism that has also been described for tumor-associated immune suppression in certain cancers is the aberrant expression of inhibitory T-cell coregulatory molecules by tumor cells. Boorjian et al (34) investigated the aberrant expression of B7-H1, B7-H3, and PD-1 T-cell coregulatory molecules in urothelial cell carcinoma, showing that that H1 molecules predict mortality after cystectomy and may represent novel diagnostic and prognostic markers.

A balance of anti-tumor effector cells and tumor-promoting suppressive and regulatory T-cells in a tumor influences not only the prognosis, but also the therapeutic impact of chemotherapy and immunotherapy (35). In NMIBC, this balance, although with different clinical outcomes, could be highlighted by CD4+:CD8+ and GrB+:CD8+ TIL ratios, which were higher in the group without recurrence. Despite the retrospective, non-randomized design of our study with possible statistical bias, we believe that these ratios could be used as a new and simple clinical immunological index for predicting recurrence, having therapeutic implications for the treatment of NMIBC patients.
